# Intrinsic and realized generation intervals in infectious-disease transmission

**DOI:** 10.1098/rspb.2015.2026

**Published:** 2015-12-22

**Authors:** David Champredon, Jonathan Dushoff

**Affiliations:** 1 School of Computational Science and Engineering, McMaster University, Hamilton, Canada L8S 4L8; 2 Department of Biology, McMaster University, Hamilton, Canada L8S 4L8

**Keywords:** generation interval, contact-tracing, epidemiological model, reproductive number

## Abstract

The generation interval is the interval between the time when an individual is infected by an infector and the time when this infector was infected. Its distribution underpins estimates of the reproductive number and hence informs public health strategies. Empirical generation-interval distributions are often derived from contact-tracing data. But linking observed generation intervals to the underlying generation interval required for modelling purposes is surprisingly not straightforward, and misspecifications can lead to incorrect estimates of the reproductive number, with the potential to misguide interventions to stop or slow an epidemic. Here, we clarify the theoretical framework for three conceptually different generation-interval distributions: the ‘intrinsic’ one typically used in mathematical models and the ‘forward’ and ‘backward’ ones typically observed from contact-tracing data, looking, respectively, forward or backward in time. We explain how the relationship between these distributions changes as an epidemic progresses and discuss how empirical generation-interval data can be used to correctly inform mathematical models.

## 1. Introduction

Much infectious disease modelling focuses on estimating the reproductive number—the number of new cases caused on average by each case. In the specific instance where the case is introduced in a fully susceptible population, we talk about the basic reproductive number 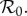 The reproductive number provides information about the disease's potential for spread and the difficulty of control. It is often thought of as a single number: an average [1] or an appropriate sort of weighted average [2]. But the reproductive number can also be thought of as a distribution across the population of possible infectors: different hosts may have different tendencies to transmit disease.

The reproductive number provides information about how a disease spreads, on the scale of disease generations. It does not, however, contain information about the population-level rate of spread (e.g. how disease incidence increases through time, which can be critical for public health interventions). Hence, another important quantity is the population-level *rate of spread*. In disease outbreaks, the rate of spread is often inferred from case-incidence reports and used to estimate the reproductive number.

The reproductive number and the rate of spread are linked by the *generation interval*—the interval between the time when an individual is infected by an infector, and the time when the infector was infected [3].

Whereas the rate of spread measures the speed of the disease at the population level, the generation interval measures speed at the individual level. It is typically inferred from contact tracing, sometimes in combination with clinical data. Like the reproductive number, the generation interval can be thought of as a single number (typically its mean) or as a distribution.

Several previous studies have investigated aspects of the generation interval. Svensson [3] made one of the earliest attempts to define a mathematical framework for the generation interval. Several authors [3,4] described a decrease in the generation interval over the course of an epidemic and it was argued that this phenomenon could be caused by competition between infectors [4]. Nishiura [5] explained, in the context of a specific epidemiological model (compartmental susceptible–infected–recovered), how observed mean generation intervals are expected to change through time and the bias this can introduce in estimating the basic reproductive number.

Generation intervals and mean generation time have also been studied in other fields, including human demography [6], bacterial population growth [7] and population genetics [8]. To our knowledge, the question of how observed generation intervals change with population dynamics has not been studied outside of epidemiology, however, possibly because other fields are more interested in relatively stable populations, and less interested in outbreaks, where such changes are likely to be important.

**Figure 1.**
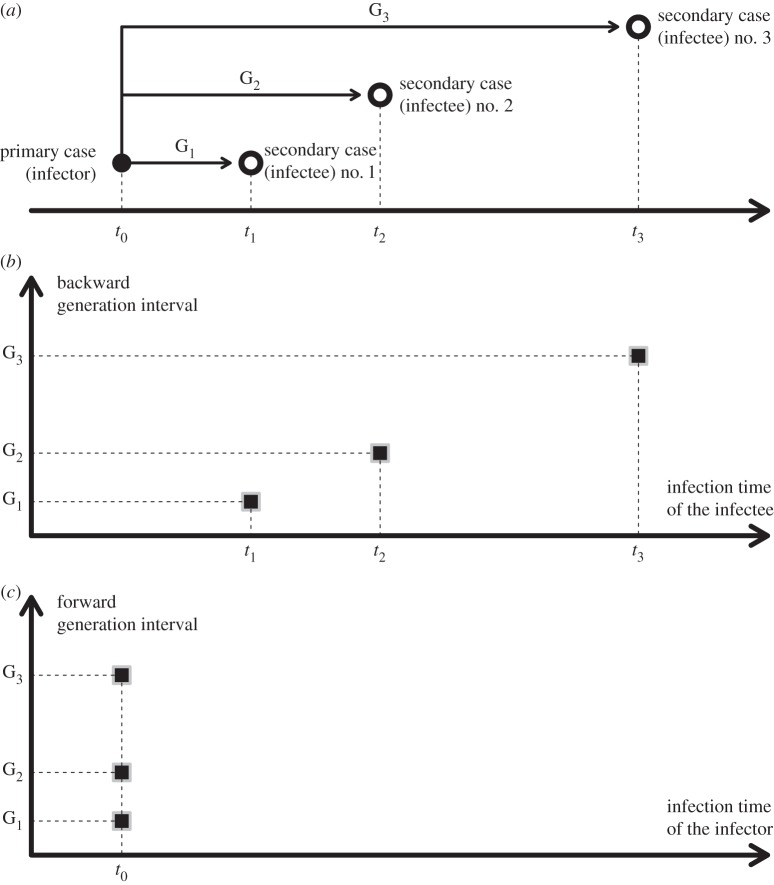
Illustration of backward and forward generation intervals. (*a*) Illustration of the example of a primary case (solid circle), infected at time *t*_0_ then infecting three other individuals (open circle), respectively, at times *t*_1_, *t*_2_ and *t*_3_. The generation intervals are defined as *G_i_* = *t_i_* − *t*_0_ for *i* = 1, 2, 3. (*b*) Plot of the backward generation intervals (black squares), that is from the infectees' point of view. There is only one backward generation interval per infectee. (*c*) Plot of the forward generation intervals (black squares) for the primary case. The *x*-axis represents the infection time of the infector, hence the three forward generation intervals are all defined at time *t*_0_.

Here, we develop a new framework to discuss generation-interval distributions and to evaluate how they change as an epidemic develops. We define an *intrinsic* generation interval whose distribution depends only on the average infectiousness of an individual at a given time after infection, and that we assume does not change as the epidemic progresses. We then investigate how this and other factors shape the distribution of *realized* generation distributions—which can either be measured *forward*, by studying who is infected by the cohort that acquires infection at a given time, or *backward*, by studying who infected a given cohort (figure 1).

Our work extends previous approaches by giving a general explanation of the temporal evolution of the full distribution of the generation interval and by confirming our theoretical results with detailed numerical simulations.

## 2. Results

### (a) Model formulation

We consider a simple and general model framework that covers a wide range of epidemiological model structures [9]. We define *S*(*t*) as the proportion of susceptible individuals in the population at time *t*, *i*(*t*) as the incidence rate—the rate at which new cases occur at time *t*—and *K*(*τ*) as the rate of secondary infections caused by an individual infected *τ* time units ago. (Note that the notation *I*(*t*) is traditionally used for disease prevalence, hence our use of lower case *i* for the incidence rate.) We can conceptually separate *K* into two components and write 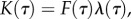 where *F*(*τ*) is the probability that an individual is infectious *τ* time units after being infected and *λ*(*τ*) is the mean infectiousness *τ* time units after an individual was infected, given that the individual is infectious at that time. Most compartmental models effectively assume that *λ*(*τ*) is a constant, but many factors could in theory affect mean infectiousness, including disease titres, how the disease spreads through the body and how active individuals are at various stages of the disease.

The number of infections occurring at time *t* caused by infectors who were themselves infected at time *s* (before *t*) is modelled as2.1
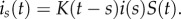


The incidence at time *t* is then given by integrating over infections caused by infectors infected at different times:2.2
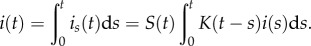


This formulation is known as the renewal equation.

In this model, the intrinsic infectiousness of a given infector, and thus the intrinsic generation interval, is described by *K*(*τ*). As we explain below, actual generation intervals that are observed (or estimated) as a disease spreads through a population do not necessarily correspond to the intrinsic generation interval.

Like several previous studies [3,5,10], we distinguish between taking the infector's point of view (looking forward in time to when secondary infections occur) or taking the infectee's (looking backward in time to when the infector was infected)—we call these *forward* and *backward* generation intervals, respectively (figure 1*c*,*b*). Hence, we define *f_s_*(*τ*) as the distribution (over *τ*) of *forward* generation intervals for infections caused *by* individuals infected at time *s* and transmitting at time *s* + *τ*. Similarly, *b_t_*(*τ*) is the distribution of *backward* generation intervals for infections *of* individuals infected at time *t* by an infector infected *τ* time units ago.

Since every generation interval has an infector and infectee, and thus a forward and backward interpretation, it is not immediately obvious why these distributions should differ. As we will see below, the distinction is owing to the way realized generation intervals change over time.

### (b) Intrinsic generation interval

From equation (2.1), we see that the intrinsic infectiousness of a given infector is simply described by *K*(*τ*). The basic reproductive number, which is the expected number infected by a single infectious individual in a totally susceptible population [1], is thus:2.3
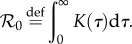


The intrinsic generation-interval *distribution* is then obtained by normalizing the intrinsic infectiousness kernel:2.4
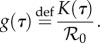


The distribution *g* is what should be estimated in order to calculate 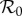 or to simulate disease spread. It is conceptually equivalent to the ‘basic’ generation time introduced by Nishiura [5].

We can thus rewrite the renewal equation (2.2) in terms of 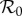 and *g*(*t*):2.5
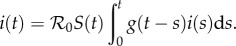


### (c) Forward generation interval

To calculate the forward generation-interval distribution, we start with the instantaneous incidence (2.1) and condition on the time *s* when the infector became infected. Thus, we replace *t* with *τ* = *t* − *s*:2.6
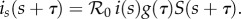


The expected number of secondary infections that will be generated per infector (*i*(*s*) = 1) is thus:2.7
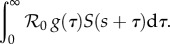


Since 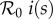 is assumed to be constant through time (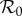 is a constant and we conditioned on time *s*), the forward generation-interval distribution for infectors infected at time *s*, *f_s_*, is proportional to 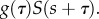 So, its definition is simply obtained by normalizing:2.8
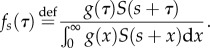


### (d) Backward generation interval

Again, using the instantaneous incidence (2.1) but now conditioning on *t*, the time when the infectee becomes infected, we have:2.9
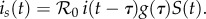


The force of infection on each susceptible individual is thus given by:2.10
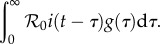


Similarly to the forward case, we see that backward generation interval is proportional to 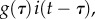 so its distribution is simply defined by normalizing:2.11
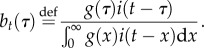


Finally, in the particular case where mean infectiousness *λ* is assumed constant over time, *K*(*τ*) is proportional to the probability *F*(*τ*), and we can write the three generation intervals directly in terms of *F*, the probability that a person is infectious at time *τ* after becoming infected:2.12
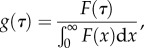
2.13
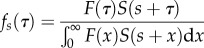
2.14
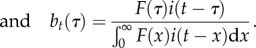


### (e) Example

In this section, we illustrate the temporal evolution of the three generation-interval distributions (intrinsic, backward and forward) described by equations (2.4), (2.11) and (2.8) with a simple epidemiological model.

**Figure 2.**
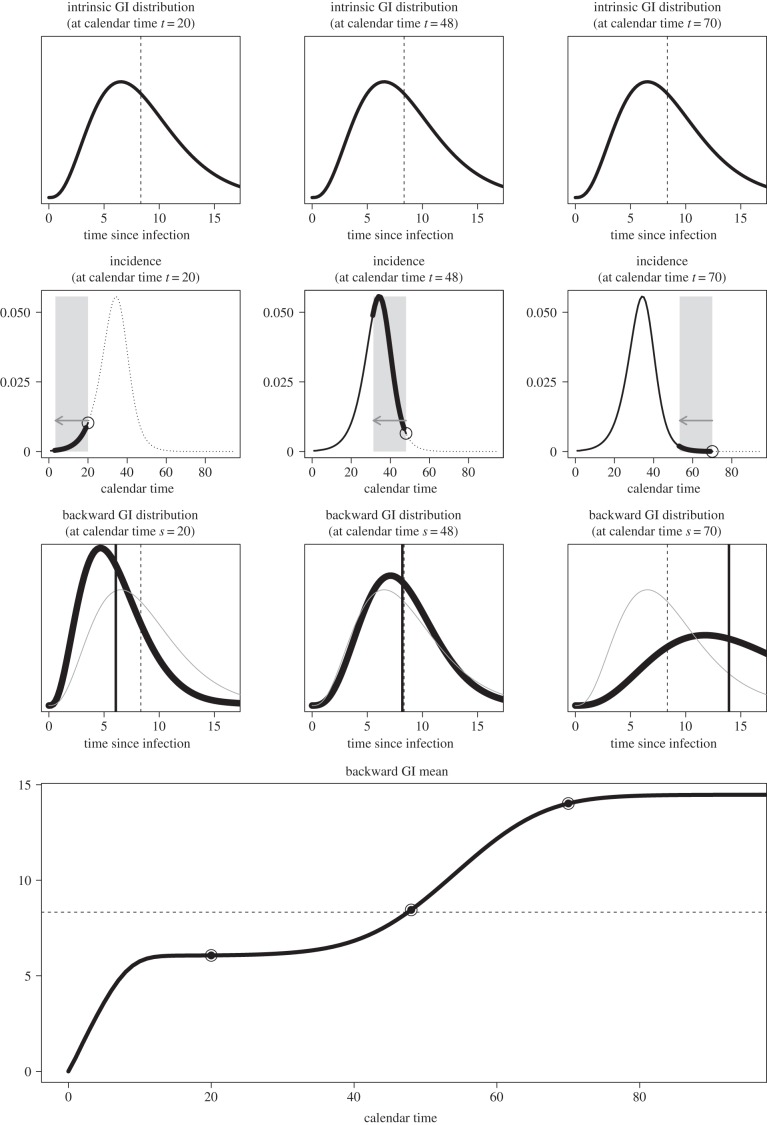
Mean backward generation interval (GI). See main text (§2e) for explanations.

**Figure 3.**
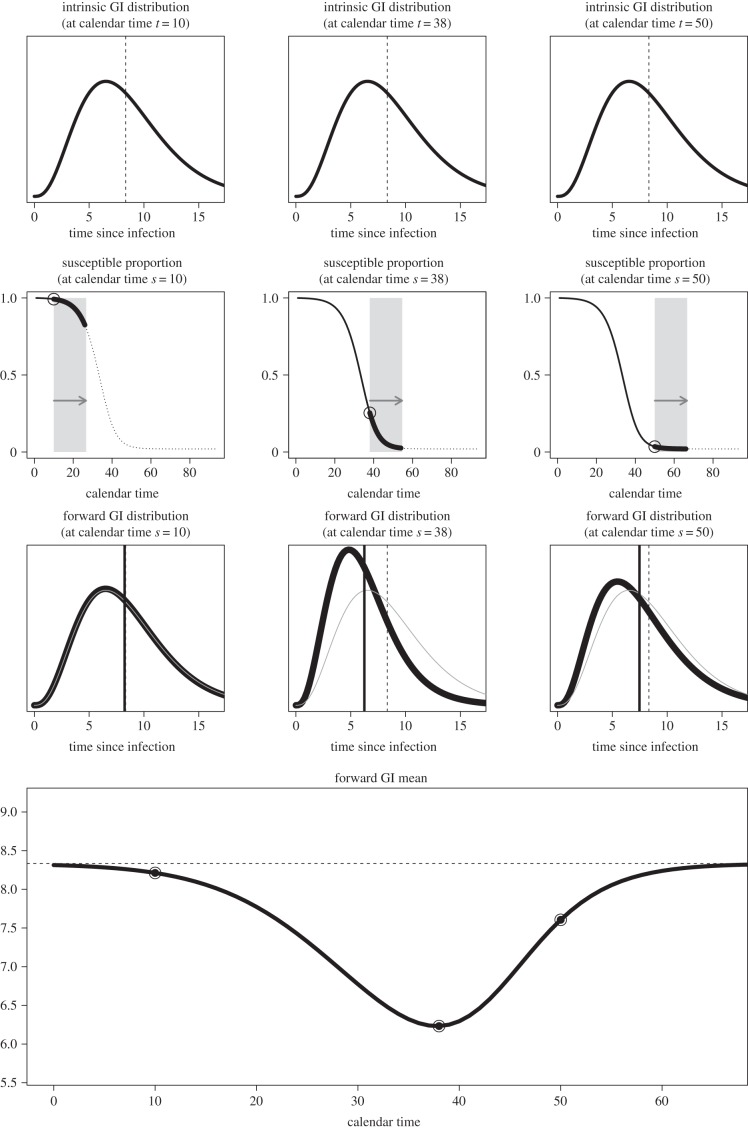
Mean forward generation interval (GI). See main text (§2e) for explanations.

In figures 2 and 3, we use the well-known SEIR compartmental model (susceptible–exposed–infectious–recovered) where we include *n*_E_ (resp. *n*_I_) exposed (resp. infectious) compartments in order to model realistic duration of latency and infectiousness with Erlang distributions (gamma distributions with integer shape parameter) [11]. We will refer to this model as an Erlang SEIR and details of this model are given in §3. This model was run with parameters: *n*_E_ = *n*_I_ = 3, 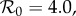 mean duration of latency and infectiousness both equal to 5 days.

Figure 2 shows how temporal variation in force of infection affects the backward generation interval *b_i_*. Left, centre and right columns represent calendar time points 20, 48 and 70 days after the start of the epidemic, respectively.

The first row shows *g*, the intrinsic generation-interval distribution. This does not change over the course of the epidemic, so the three figures on the first row are the same. The vertical dashed line at 8.3 days represents the mean of the distribution.

The second row shows the incidence curve *i*. The dotted curve is the incidence over the course of the whole epidemic. The open circles shows the current calendar time. The bold curves and the shaded areas illustrate that we look backward to multiply the intrinsic generation distribution by the incidence curve shown to obtain the backward distribution (the width of the shaded areas matches the width of the curves shown in (*a*). The grey arrows show the direction of integration, here looking backward from the current time. The third row depicts the backward generation-interval distribution (bold curve, with mean shown by a vertical bold line) resulting from equation (2.11), which is the product of bold curves from the first (intrinsic generation interval) and second row (time-reversed incidence). The intrinsic generation interval (grey curve, mean shown by a vertical grey line) is shown for comparison. Finally, the last row illustrates how the mean backward generation interval changes through time throughout the epidemic. The horizontal dashed line represents the mean intrinsic generation interval. The three circles represent the calendar time points (20, 48 and 70 days) chosen for the illustrations in the second and third rows.

Similarly, figure 3 shows how temporal variation in the susceptible population affects forward generation interval *f_s_*. Just as changes in the backward generation interval are explained by patterns of change in incidence, changes in the forward generation interval are explained by patterns of change in the proportion susceptible. Calendar time points were chosen to be 10, 38 and 50 days in this case. Animated versions of figures 2 and 3 are provided in the electronic supplementary material (movie_GI.gif).

The backward generation-interval distribution differs significantly from the intrinsic one, its mean increasing monotonically from 0 to values much larger than the mean intrinsic generation interval. The backward generation time is seen from the point of view of a susceptible: who is likely to infect them? Early in the epidemic, when the number of infectious individuals is increasing, the backward generation time tends to be short, because relatively more currently infectious individuals were infected recently. Similarly, when the epidemic is declining, there will be relatively fewer infectious individuals infected recently, tending to increase the backward generation time.

The forward generation interval is seen from the point of view of the infector: when are they likely to infect somebody? Since the number of susceptibles decreases throughout a single epidemic outbreak, there will always be relatively more susceptibles available soon after infection than later, so the mean forward generation time will always be less than the intrinsic generation time. Early and late in the epidemic, however, the number of susceptibles changes slowly, so the forward generation time is approximately the same as the intrinsic generation interval (figure 3). The shorter generation interval in the middle of the epidemic may seem counterintuitive: why do infections happen faster when susceptibles are being depleted rapidly? The answer is that we calculate the generation-interval distribution *conditional on an infection occurring*. As the number of susceptibles decreases, the number of infections per infectious individual goes down, but the infections that do happen tend to happen faster because the *relative* number of susceptibles is higher in the near future than later on (middle panel of figure 3, at calendar time 38).

Our example above is constructed with a particular value of 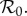 In figure S1 in the electronic supplementary material, we show how the mean generation intervals change through time for a range of 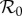 values. All else being equal, higher 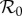 leads to faster epidemics, and sharper deviations of both forward and backward generation intervals from the intrinsic generation-interval distribution *g*. Note that this figure is very similar to fig. 3 in [5], but with the important difference that here, we explicitly mark the epidemic endpoints (solid circles in figure S1) to illustrate the actual deviations that can be experienced in practice.

### (f) Comparison with simulations

We compare the analytical formulations of both forward (2.13) and backward (2.14) generation intervals with stochastic simulations in the Erlang SEIR framework, assuming a constant infectiousness *λ*.

**Figure 4.**
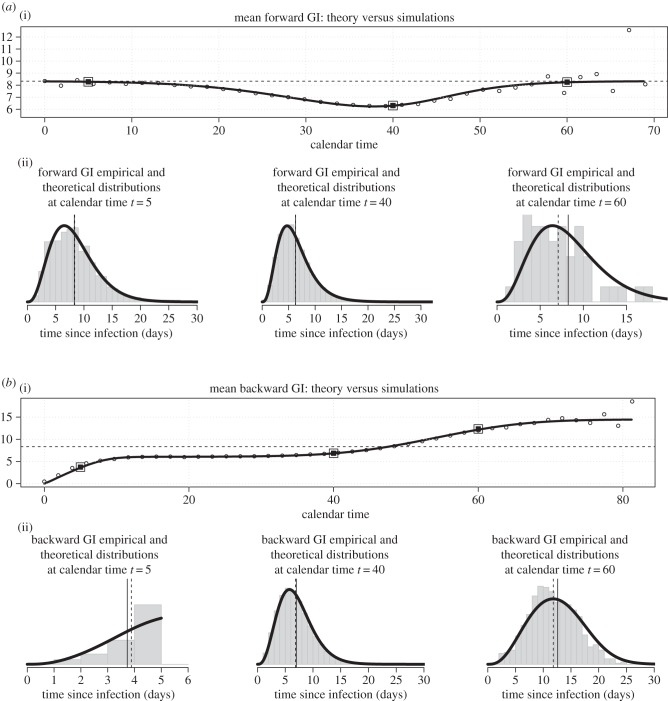
Mean generation intervals: theory versus simulations. Numerical validation of forward and backward generation-interval distributions. *a*(i) The thick line is the mean of the forward generation interval obtained by integrating equation (2.13). The open circles represent the mean of the forward generation intervals from stochastic simulations. The horizontal dashed line depicts the mean intrinsic generation interval. The three squares show the calendar times chosen for the distribution in the second panel. *a*(ii) Empirical (grey histogram) and theoretical (black line) forward generation-interval distribution at calendar times 5, 40 and 60 days. The solid (resp. dashed) vertical line represents the mean of the theoretical (resp. empirical) distribution. Parts *b*(i,ii) represent the same quantities as the first and second panels, but for the backward generation interval using equation (2.14). Model parameters: 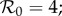 *n*_E_ = *n*_I_ = 3; mean latency and mean infectious duration both equal 5 days; Monte Carlo iterations = 30; population size = 25 000.

Figure 4 shows good agreement of the mean generation intervals (both forward and backward) between the stochastic simulations (using a Gillespie algorithm [12], see §3) and the numerical solutions of equations (2.13) and (2.14).

## 3. Material and methods

### (a) Compartmental model

**Figure 5.**
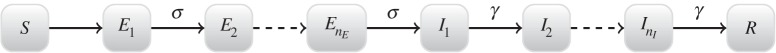
Erlang SEIR model.

To estimate generation-interval distributions for our examples, we used numerical simulations with a flexible compartmental model: a classical SEIR model (susceptible–exposed–infectious–recovered) with *n*_E_ (resp. *n*_I_) sub-compartments for the exposed (resp. infectious) state [13,14] (figure 5). This modelling framework implicitly specifies Erlang-distributed (i.e. gamma distribution with integer shape parameter) duration of latency and infectiousness that can reasonably approximate real epidemiological observations [11]. A deterministic formulation of this model is given by a system of differential equations. Let *S* be the proportion of susceptible individuals in the whole population; *E_k_* the proportion of individuals in the *k*th compartment of latency (i.e. infected but not infectious yet); *I_k_* the proportion of individuals in the *k*th compartment of infectiousness; *β* the constant effective contact rate; *σ* the average rate of progression from one latency stage to the next; *γ* the average rate of progression from one infectious stage to the next. The model is given by the system of equations (3.1):3.1*a*
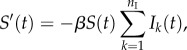
3.1*b*
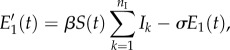
3.1*c*
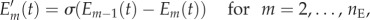
3.1*d*
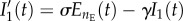
3.1*e*
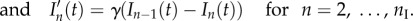
Similarly, a system of differential equations defines the probability of residency in a given latent or infectious state for individuals infected at a fixed time *s*. We define *L_k_*(*τ*) as the probability that an individual infected *τ* time units ago is in the *k*th latent stage *E_k_*, and *F_k_*(*τ*) as the probability that an individual infected *τ* time units ago is in the *k*th infectious stage *I_k_*. We have:3.2*a*
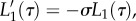
3.2*b*
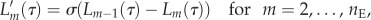
3.2*c*
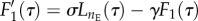
3.2*d*
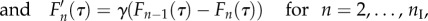
with the initial conditions 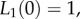 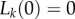 for all 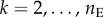 and 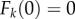 for all 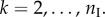

We solved both systems (3.1) and (3.2) numerically using the **lsoda** method from the **R** [15] package **deSolve** [16] v. 1.11.

### (b) Stochastic simulations

We validated the results from our deterministic model (3.1) by implementing a discrete-state stochastic version of this model using an exact Gillespie algorithm [12]. Briefly, this algorithm simulates exponentially distributed event times for progression from one state to the next one (e.g. from susceptible (*S*) to exposed (*E*)). Both the intensity and event type frequency depend on the rates defined in (3.1). We extend the classical Gillespie algorithm by identifying every individual in the simulation. Hence we can keep track of the generation intervals at pre-specified times both from the infector (forward) and the infectee (backward) viewpoints. The outputs of interest from a simulation are pairs of generation interval (forward or backward) and calendar time (time elapsed since the start of the epidemic). Simulated generation intervals are aggregated and averaged in 1-day time buckets. A detailed description of this algorithm is given in electronic supplementary material, algorithm 1.

## 4. Practical implication

During the early phase of a pathogen outbreak, generation interval information is strongly ‘censored’, since a relatively large proportion of infections are still ongoing. This information cannot be used to reliably estimate forward generation intervals, but can estimate backward generation intervals (or it may simply be lumped, which has a similar effect to using backward intervals). A naive—but common—approach is to use the mean (and sometimes the variance) of this data to inform the intrinsic generation interval distribution *g* of a mathematical model (e.g. used for forecasting). This method will lead to a systematic bias: since shorter generation intervals are more likely to have concluded (and thus be observed), the mean generation interval will be systematically underestimated [5]. An alternative approach is to fit the backward generation interval distribution *b* of the model (obtained with equation (2.11)) to the backward contact-tracing data at each available calendar time. As an approximation to fitting the whole distribution, one can aim to fit both the mean and variance of the backward generation interval distribution to the data.

**Figure 6.**
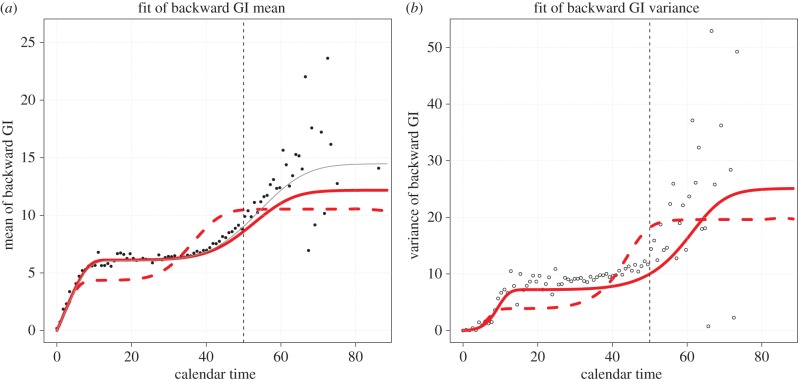
Comparison between fitting the backward (*b*) or intrinsic (*g*) generation interval distribution of an Erlang SEIR model to synthetic data. Model parameters used to generate the data were 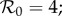 *n*_E_ = *n*_I_ = 3; mean latency and mean infectious duration both equal to 5 days; population size at 25 000. (*a*) Fit to the mean of the backward generation interval distribution. Solid black circles are the simulated backward generation intervals data. The red solid thick curve is the fitted mean backward generation interval *b* from the Erlang SEIR model to the mean backward generation intervals data. The red dashed thick curve is the fitted mean backward generation interval *b* when fitting (naively) the *intrinsic* distribution *g* from the same Erlang SEIR model to the backward generation intervals data. The thin grey curve is *b* when using the ‘true’ parameter values that generated the simulated data. (*b*) Fit to the variance of the backward generation interval distribution. Open circles represent the simulated data. The red thick solid line is the variance of the fitted distribution *b* when fitting *b* to the simulated backward generation intervals data. The red thick dashed line represents the variance of the fitted distribution *b* when (naively) fitting the intrinsic distribution *g* to the simulated backward generation intervals data. Only the points to the left of the vertical dashed line (at calendar time 50) were used in both fits. An approximate Bayesian computation method with 1000 iterations was performed for both fits. (Online version in colour.)

The backward generation interval fit to the mean and variance is illustrated in figure 6, where contact-tracing data were simulated from an Erlang SEIR model. The potential pitfalls of naively fitting, without recognizing the difference between the intrinsic generation interval distribution *g* and the observed backward interval *b*, are also shown (dashed lines): the resulting mean and variance of observed intervals are a notably poorer match to the data.

Note that, depending on the model complexity, the minimization problem can be high dimensional and there might be identifiability issues between parameters. Also, when the number of data points is small (very first days of the outbreak), fitting may be challenging because the mean of the backward generation interval distribution *b* is very insensitive to model parameters (figure S1*a*).

This example serves as a simple illustration for an important point: data from contact-tracing can provide misleading information about a pathogen's underlying intrinsic generation interval *g*. The factors that underlie how the realized forward and backward generation intervals change through time should be taken into account when evaluating observed intervals. Future work on constructing a more elaborate and robust statistical framework to perform such fit is warranted.

## 5. Discussion

Conceptually, there are three generation-interval distributions (table 1). We called the first ‘intrinsic’ generation-interval distribution, which defines theoretically the disease transmission process. This is the distribution typically used in mathematical models, such as in the well-known renewal equation (2.2), and is often assumed invariant with respect to time. Variation in intrinsic generation interval, if it occurs, is driven by changes in the biological or social processes underlying disease transmission, e.g. quarantines or social-distancing practices, but not by the spread of disease *per se*. The other two generation-interval distributions are typically obtained by *observing* the actual infection time differences between the infector and its infectee. If the point of view is from the infectee, then there is only one interval to consider and this defines the so-called ‘backward’ generation interval. If we take the infector's viewpoint, then there are potentially several generation intervals (because the infector could have infected several individuals) and this defines the ‘forward’ generation interval (figure 1*c*). In other words, if we believe that generation intervals are drawn from their respective distribution (i.e. *b* and *f* with our notations), then the backward generation interval we get in the first place is a single draw, whereas the latter represents several draws.

**Table 1.** The three generation-interval distributions.

generation-interval distribution type	notation	usage	defining equation
intrinsic	*g*	mathematical modelling	(2.4)
backward	*b*	observed when a cohort is investigated by looking backward in time to see who infected each individual	(2.11)
forward	*f*	observed when a cohort is tracked forward in time to see whom individuals infect	(2.8)

We have developed a theoretical framework that explains the temporal variation of both backward and forward generation-interval distributions. We confirm the findings from Nishiura [5] that were derived for mean generation intervals in the context of exponential growth of incidence. We extend their interpretability to the whole generation-interval distribution (not the mean only) in a general modelling framework (no exponential growth assumption). In particular, our interpretation does not involve the concept of competition between infectors [4].

Our theoretical results were confirmed with numerical simulations, using an Erlang SEIR compartmental model. Note that other sorts of models should work equally well, as long as it is possible to derive analytically or numerically the proportion of susceptible individuals *S*, the incidence *i* and the probability *F* to be infectious *τ* time units after being infected.

As noted by previous studies [3–5,10], the temporal shape of the mean backward generation interval in figure 2 has important modelling consequences. Indeed, the mean backward generation interval can remain significantly below (resp. above) the mean intrinsic generation interval early (resp. late) in the epidemic. So, estimates of the generation intervals obtained by contact tracing can underestimate (when observed too early) or overestimate (when observed too late) the mean intrinsic generation interval. This is related to the problem of ‘length-biased sampling’ [17]. Put simply, generation intervals measured through contact-tracing may be a biased estimate of the intrinsic generation interval. In particular, if estimates of *b* are used to estimate *g* early in an epidemic, the length of the generation interval is likely to be underestimated. This effect is more pronounced when the reproductive number of the epidemic considered is large (figure S1).

An important application of generation intervals is the estimation of the basic reproductive number 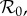 which is in turn used for various public health decisions. The *intrinsic* generation-interval distribution *g* is the link between the observed growth rate (incidence data) and 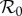 [18]. If *g* is systematically underestimated, as discussed above, 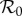 is likely to be underestimated as well (see [5] for an example illustrating this issue on Dutch influenza data).

Our work suggests a possible methodology to correct for these potential pitfalls: if it is possible to derive (analytically or numerically) from the mathematical model either the intrinsic generation-interval distribution *g* or the probability *F* of being infectious after a given duration from the infection time, then modellers can derive the backward (or forward) generation-interval distribution from equations (2.11) and (2.14), and fit this distribution (and not *g*) to the relevant contact-tracing data.

There are some limitations to our work. First, while we consider generation intervals, in practice serial intervals (the interval between *symptom onset* in the infector and symptom onset in the infectee) are easier to observe for many diseases. Serial intervals are less tractable theoretically and in general do not have the same distribution as generation intervals. However, in some settings, their distribution can be strongly correlated or even identical [3,19]. Second, our theoretical framework relies on the assumption of homogeneous mixing. Although this is a common assumption, heterogeneity is often very important in practice (see [20] for example), and could affect the patterns found here. Third, we do not account for the possibility that mixing rates or the course of infection change through time, for example owing to seasonality, awareness of the epidemic, or medical intervention. Like earlier authors, we focus on the intrinsic dynamics of the disease system. Fourth, a robust, statistically based method to infer model parameters associated to generation intervals from observable data available early in an epidemic is still needed. Statistical methods have been proposed to estimate intrinsic generation intervals (e.g. [21–23]), but further work is necessary to extend these methods to our framework, for example constructing estimation methods directly using the backward generation interval distribution in the context of missing data. Establishing a link between our framework and the serial interval (the most likely observable quantity) is also warranted.

Informing the generation-interval distribution of a mathematical model from contact-tracing data is not straightforward, and a naive approach can lead to spurious epidemiological projections from the model. Extending previous work, our study provides a clear and coherent theoretical framework to understand and assess the differences between three conceptually distinct generation-interval distributions. Future work should consider building statistical tools leveraging our study on real contact-tracing data.

## Supplementary Material

Figure S1

## Supplementary Material

Algorithm for Erlang SEIR model

## Supplementary Material

Distributions temporal evolution

## Data accessibility

The computer code that was used to generate the numerical results and figures in this study is available on Dryad at http://dx.doi.org/10.5061/dryad.4dd3s.
