# Supplementary material for: Intrinsic and realized generation intervals in infectious-disease transmission
Source: Proc Biol Sci. 2015 Dec 22;282(1821):20152026. doi: 10.1098/rspb.2015.2026 (PMC4707754; doi:10.1098/rspb.2015.2026)
Supplement: Figure S1 [file rspb20152026supp1.pdf]

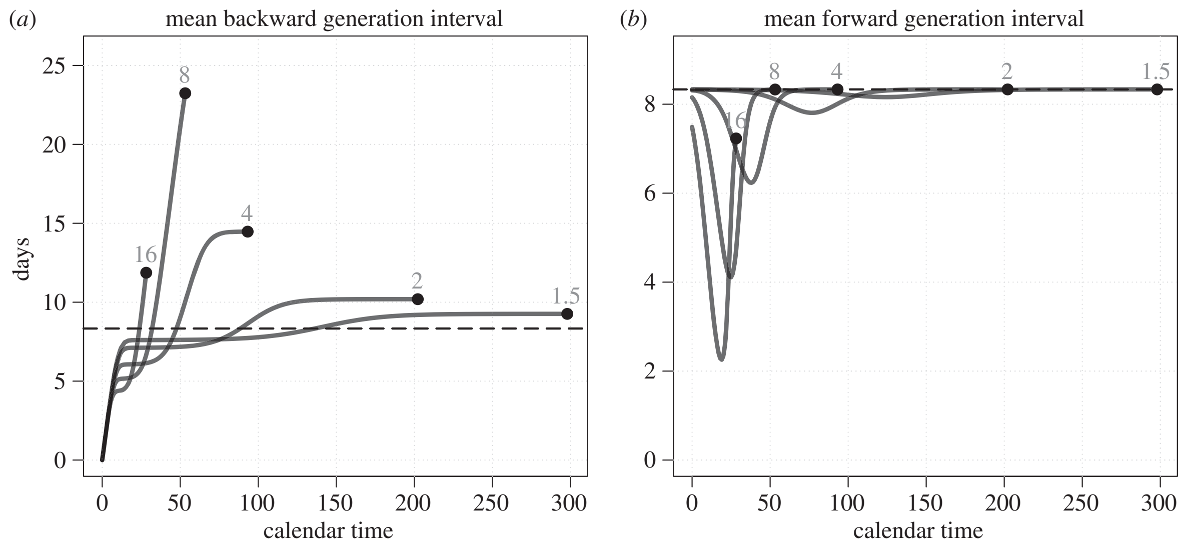

Figure S1. Temporal evolution of mean (*a*) backward and (*b*) forward generation intervals for different values of $\mathcal{R}_0$. Curves were integrated until the time of last incident case (solid circles) and the value of $\mathcal{R}_0$ is indicated at this endpoint (grey numbers). The horizontal dashed line is the mean intrinsic generation interval.
