# Supplementary material for: Intrinsic and realized generation intervals in infectious-disease transmission
Source: Proc Biol Sci. 2015 Dec 22;282(1821):20152026. doi: 10.1098/rspb.2015.2026 (PMC4707754; doi:10.1098/rspb.2015.2026)
Supplement: Algorithm for Erlang SEIR model [file rspb20152026supp2.pdf]

Algorithm 1: Simplified Gillespie algorithm for the Erlang SEIR model

Input: Time horizon T, contact rate β, number of I (resp. E) compartments n_I (resp. n_E), mean residency time in any I compartments $1/\gamma$, mean residency time in any E compartments $1/\sigma$, initial number of infectious individuals i_0, total population N.

$t \leftarrow 0$
$S[0] \leftarrow N - i_0$
$E_k[0] \leftarrow 0$ for all $k > 0$
$I_1[0] \leftarrow i_0$
$I_k[0] \leftarrow 0$ for all $k > 1$

while $t < T$ **do**
  `/* Draw the next event time` τ `*/`
  $\lambda_S \leftarrow \beta S[t] \sum_{k=1}^{n_I} I_k[t]/N$
  $\lambda_E \leftarrow \frac{\sigma}{n_E} \sum_{k=1}^{n_E} E_k[t]$
  $\lambda_I \leftarrow \frac{\gamma}{n_I} \sum_{k=1}^{n_I} I_k[t]$
  $\lambda \leftarrow \lambda_S + \lambda_E + \lambda_I$
  $\tau \sim \mathrm{Exp}(\lambda)$
  `/* Draw the next event type */`
  $u \sim \mathrm{Uniform}(0, \lambda)$
  if $u < \lambda_S$ **then**
    `/* event type is new infection */`
    Pick randomly a new infectee among $S[t]$: individual a
    Set infection time for individual a at $t + \tau$
    Pick randomly its infector among $I_1[t], ..., I_{n_I}[t]$: individual c
    `/* calculate generation interval */`
    $G \leftarrow t + \tau - \mathrm{time.acquisition}(c)$
    `/* backward generation interval for individual a (infectee) */`
    $b(a) \leftarrow G$
    `/* forward generation interval for individual c (infector) */`
    $f(c) \leftarrow \mathrm{vector}(f(c), G)$
  else
    `/* event type is not infection */`
    Pick randomly individual in ad hoc compartment (based on u value)
    Move this individual to the next compartment
  end

  $t \leftarrow t + \tau$
end
