# Supplementary figures and images for: Intrinsic and realized generation intervals in infectious-disease transmission
Source: Proc Biol Sci. 2015 Dec 22;282(1821):20152026. doi: 10.1098/rspb.2015.2026 (PMC4707754; doi:10.1098/rspb.2015.2026)

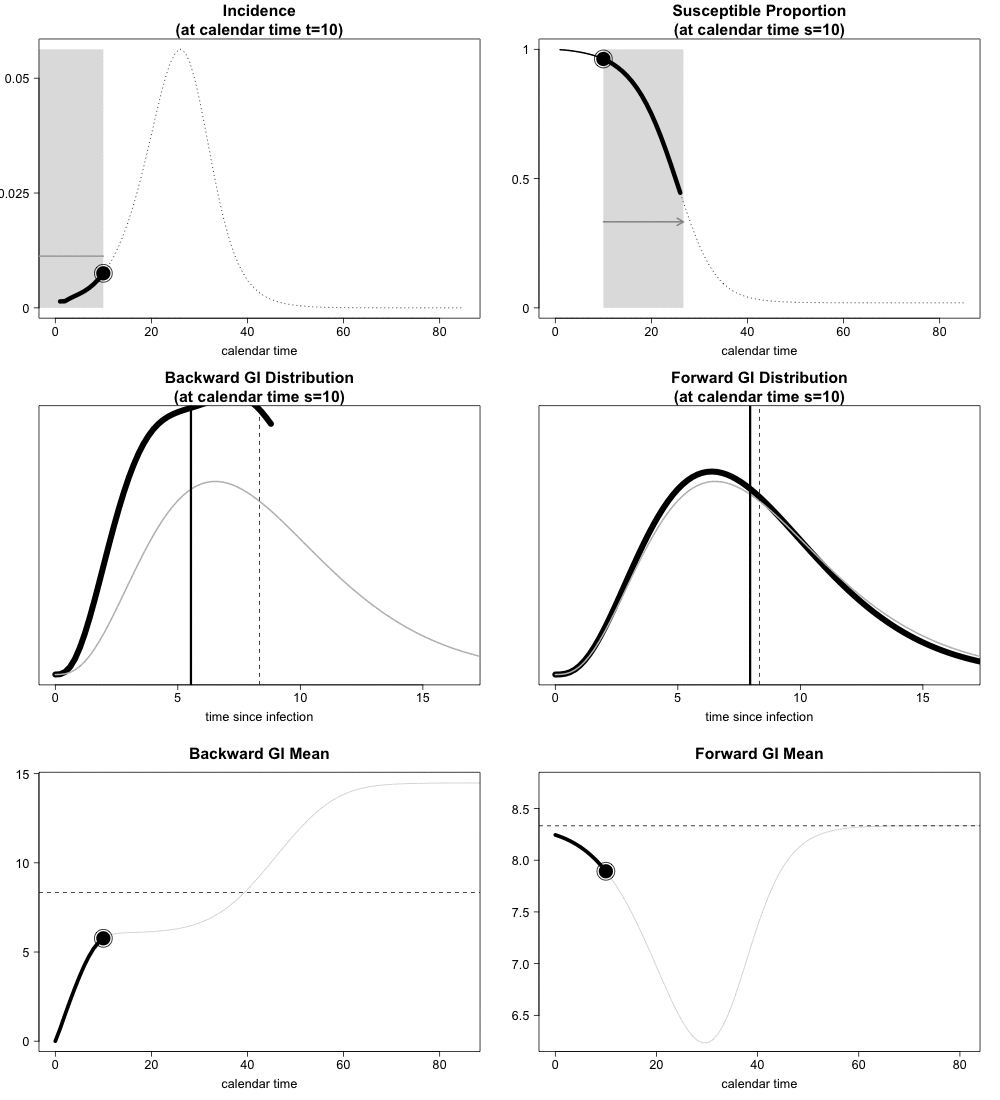

Supplement: Distributions temporal evolution [file rspb20152026supp3.gif]
